# New perspectives and issues in industrial policy for sustainable development: from developmental and entrepreneurial to environmental state

**DOI:** 10.1007/s43253-023-00100-2

**Published:** 2023-05-24

**Authors:** Ioanna Kastelli, Lukasz Mamica, Keun Lee

**Affiliations:** 1 National Technical University of Athens, Athens, Greece; 2 Hellenic Open University, Patras, Greece; 3 Krakow University of Economics, Rakowicka 27, 31-510 Kraków, Poland; 4 Seoul National University, Seoul, Korea; 5 College of Business and Economics, University of Johannesburg, Johannesburg, South Africa; 6 CIFAR (IEP), Toronto, Canada

**Keywords:** Industrial policy, Sustainable development, Capability failure, Institutional variety, Green growth, Industrial transformation, O25, O33, Q01

## Abstract

The increasingly acute consequences of the climate crisis, the COVID-19 pandemic, and the energy crisis have put industrial policy back. The papers in this issue examine how different countries implement industrial policy for sustainable development from a variety of perspectives. A successful transition to sustainable development seems to require not only the mix of carrots and sticks but also a right mix of creation versus destruction, as in the case of the creation of renewable businesses and the destruction of fossil-fuel businesses. Furthermore, because institutional diversity and the risk of capture can result in very distinct economic, social, and environmental effects, consideration of heterogeneity at the country and sector levels and coordination of vested interests are essential ingredients for sustainable industrial policies, as shown by the case of industrial policy in France and the two industry cases in India. By contrast, the Amazon Fund case is indicative of the three success elements: multi-stakeholder governance, pay-for-performance funding, and non-reimbursable project financing. These three elements can be summarized as local ownership and accountable governance, provided with both carrots and sticks. The problematic case of urban development driven by the oil industry in Ghana can be criticized in terms of the lack of local ownership of the oil industry, which has led to all rents being monopolized by the absentee class. By comparison, the mixed success of cases of industrial symbiosis in Uganda is attributed to the lack of effective carrots. In sum, industrial policy for sustainable development requires handling well all three types of failure, namely, market, system, and capability failures, because it necessitates building capabilities of involved actors and coordinating actions of agents, in addition to providing optimal incentives to reflect externalities of global public goods. Overall, the shifting focus of industrial policy is consistent with the shift of the role of the state, from developmental to entrepreneurial, and finally to environmental state.

## Introduction

The recent pandemic crisis, the current Ukrainian war in Europe, and its global socio-economic effects, as well as the long-overdue actions to mitigate climate change, remind us that significant risks arise from the combination of adverse and disruptive events related to environmental sustainability, pandemics, socio-economic inequalities, and geo-political turbulence. Since 2015, the transformation of the world towards sustainability in line with the 2030 Agenda is linked to specific sustainable development goals (SDGs) reflecting the required combination of environmental, social, and economic dimensions. In this context, industrial policy links directly to some of the SDGs but can influence to a different extent all goals.

Although industrial policy was present and evolving in diverse names and variations, the revamping of interest on industrial policy in the aftermaths of the 2008 financial crisis started shifting towards encompassing long-term qualitative transformations to increase prosperity and living standards and advance economic competitiveness and growth but at the same time safeguarding the environment by raising resource productivity and minimizing the negative environmental impact of industrial activities (UNIDO 2022). Thus, this paper proposes and discusses a new vision of industrial policy that can be defined as industrialization that drives development along the three aspects of inclusion, sustainability, and resilience. In that sense, industrial development is to create shared prosperity by offering equal opportunities and equitable distribution of benefits to all. The combined goal of advancing economic competitiveness and safeguarding the environment is possible by decoupling the prosperity generated by industrial activities from excessive natural resource use and negative environmental impacts (UNIDO 2022). In this light, this new industrial policy is different from the traditional definition by Johnson (1982), namely, as any policy that improves the structure of a domestic industry to enhance a country’s international competitiveness.

Recent studies tracking the development of relevant indicators for SDGs against gross domestic product per person in different regions have shown that there is a limit to benign growth fulfilling human well-being, that the cost of not doing anything and the amount of effort needed to meet sustainability goals are huge, and that societies must accelerate sustainability transformations for an equitable future on a finite planet (Collste et al. 2021; Forster et al. 2020; IPCC 2018). Thus, this paper adopts an argument for government activism that goes beyond market and system failures and highlights the role of capability failure (Lee 2013). We also argue that structural change as a long-term process is highly heterogeneous across countries and regions in terms of capabilities, opportunities, needs, and ways of reaction to emerging problems. This underscores the importance of adopting different policy tools over time or in different contexts. It also raises the political issue of inclusive sustainable development, because sustainability issues are global, but the range of possibilities differs substantially across stakeholders.

Following this line, we highlight specific elements that need to be addressed for sustainable industrial development and should be part of the current debate on industrial policy. They are (i) the role of production and learning as a process embedded in production structures; (ii) the need to consider the demand side as contributing to industrial transformation for sustainable development; (iii) the interplay of productive and technological aspects with institutional change and the role of institutional diversity; (iv) the political economy dynamics entailed in the process of industrial transformation toward sustainable development; (v) the need for coordination across policies, instruments, and actors and the importance of a new type of public management; and (vi) the required shift of evaluation criteria of industrial policy beyond productivity and competitiveness towards encompassing ecological, social, and economic objectives for the long-term rise of life-standards. Hence, we put forward a novel way of reasoning, linking industrial policy to a political process that ideally should bring up a consensus on a mission for sustainable development. For this consensus to be legitimate, the involvement of all stakeholders is required because a multitude of contradictions and controversies need to be overcome. Industrial policy for sustainable development implies a system of policies to foster a transition to a more sustainable mode of economic activity.

Therefore, one of the foci in this paper is to contrast new industrial policy with traditional industrial policy in terms of their goals and tools. Moreover, given the existing concern about the cases of failures and inefficiency of traditional industrial policy, this paper also looks at the issue of what would be the conditions for the success (or failure) of new industrial policy. This contrast and comparison involve eventually the role of the state. The emergence of new industrial policy is consistent with a shift of the role of the state to what should be called the “environmental state” promoting sustainable development at a global scale beyond the earlier roles such as developmental state (Johnson 1982) or entrepreneurial state (Mazzucato 2013). In this context, the orchestration of policy initiatives by the state should encompass building productive, technological, and organizational capabilities, integrating the demand aspect and institutional transformation, mobilizing and directing financial resources, to meet SDGs.

To elaborate further on this discussion, we have launched a call for papers for this special issue of the Review of Evolutionary Political Economy on “Industrial Policy for Sustainable Development” and accepted six contributions from distinguished researchers that identify with and share many of the issues raised above. The contribution of this project is twofold. It links elements that have been addressed in the context of the renewal of interest in industrial policy with industrial transformation required for tackling sustainability challenges. Furthermore, it extends this discussion beyond European and emerging economies to less developed or African countries, showing the important differences in implemented strategies so far and revealing that although sustainability challenges are global, there is an extreme heterogeneity in structural and institutional characteristics as well as political economy dynamics with substantial variation in the implementation of industrial policy, resulting in very distinct economic, social, and environmental effects. Industrial policy for sustainable development should then mediate a “creative destruction” force that involves emergent and incumbent stakeholders and a risk of capture that eventually hinders inclusion and just transition.

This paper is structured as follows. The following section provides a brief overview of the evolution of the literature on industrial policy with specific reference to the arguments for government activism and several types of failures. The third section discusses new elements that should be integrated into the analytical framework of industrial policy. The fourth section provides a brief overview of the papers in this special issue. The final section concludes with some summary and open questions for future research.

## Evolution of the literature on industrial policy

### A brief overview

One of the earliest and classical works on industrial policy is Johnson (1982), which attributed the success of the Japanese economy since the 1970s to the critical role of the government, in particular the Ministry of International Trade and Investment. The history of capitalism indicates that variants of industrial policies existed in diverse countries, such as the UK from the 14th to the eighteenth centuries, the USA and Germany in the nineteenth century, Japan in the late nineteenth century, and South Korea and Taiwan in the late twentieth century (Cimoli et al. 2009; Wade 1990). Specifically, as South Korea followed Japan to achieve a rapid economic catch-up, Amsden (1989) analyzed the Korean case from a similar perspective and attributed the Korean miracle to the policy of “getting prices wrong rather than right” by the government where price manipulation by the government generated rents for specific industries which acted as incentives for entry by big businesses or chaebol firms. Thus, the role of the state in East Asia has been considered a “developmental state” (Thurbon 2014; Johnson 1982).

Despite its recognized contribution to the East Asian miracle (World Bank 1993), industrial policy has been regarded as a taboo in the mainstream economics literature since the arrival of the WTO regime in the midst of the neoliberal order in the global economy. However, the 2008 global financial crisis resulted in the revival of industrial policy, which was initiated by a notable volume of Stiglitz and Lin (2013) and Mazzucato (2011). In particular, Mazzucato (2011) re-invented the role of the state, in the name of the entrepreneurial state, even in the advanced economies.

Calling it a revival might not be precise because industrial policy has continued to evolve in diverse names and variations, such as innovation policy (Edler and Fagerberg 2017; Soete 2007), industrial innovation policy (Nelson and Langlois 1983), and mission-oriented innovation policy (Mazzucato 2018). Such evolution is not surprising, given that although there have been many cases where industrial policy has failed, no latecomer economy has achieved sustained catch-up without relying on some form of industrial policy or public intervention. In recent decades, however, its meaning has changed and evolved to deal with the pressing concerns of the twenty-first century, including environmental degradation and sustainable development (Criscuolo et al. 2022; Radošević et al. 2017), which indicates the possibility of “environmental state.”

Recently, the role of the government or the state has received a renewed attention as a new trend toward de-globalization has been triggered by a series of events, including the 2008 global financial crisis, the rise in US–China tensions since 2019, the COVID-19 pandemic, and the Russia–Ukraine war. At present, we are witnessing the increasing and changing role of national governments not only in developing but also in developed economies. For instance, in their article on policy matrixes for inclusive growth, Rodrik and Stantcheva (2021) argue that governments should now intervene during the production stage using various means, including industrial policy, besides either the pre- or post-production stages using such means as education or welfare schemes.^1^ Proponents of this approach argue that if a government fails to intervene during the production stage and successfully promote the international competitiveness of its domestic industries, its firms may fail and workers will lose their jobs, placing a burden on welfare systems.

### Three types of failures and re-interpreting the empirical evidence

A classical argument for government activism, particularly industrial policy, has been made in the context of market failure. The new structural economics of Lin (2011) as well as the initiatives put forward by Cimoli et al. (2009) argue for government activism. Governments are advised to promote infant industries and facilitate industrial upgrade and diversification, which are justified by information and coordination failure that can be regarded as broadly defined market failure. The source of market failure is the fact that knowledge is a public good. Industrial policy is then justified due to possible underinvestment in learning when there are failures in the capital and risk markets, as well as spillover in learning. From this perspective, the actual amount of R&D is often less than the optimal amount that would prevail without market failure. Therefore, government subsidies to support R&D are suggested. In sum, industrial policy is defined as closing the knowledge gap (Stiglitz and Greenwald 2014).

Another view that supports a proactive government is the system failure view of neo-Schumpeterian economics. Specifically, the concept of the national innovation system of Nelson (1993) and Lundvall (1992) calls for government activism with a different basis from that of the market failure view. Metcalfe (2005) argues that the process of innovation depends on the emergence and success of innovation systems connecting the various actors engaged in the process, and that the need for government activism arises because effective interaction among the actors does not exist naturally but has to be constructed (Bergek et al. 2008; Dodgson et al. 2011). System failure arises due to cognitive distance among these actors and/or tacitness of knowledge, resulting in cognition failure (Nooteboom 2009). In this situation, the government may intervene to set the framework conditions in which innovation systems can better self-organize across a range of economic activities.

In the meantime, there is a need to re-assess the aforementioned views regarding whether they can be considered an effective rationale for the degrees and forms of government activism in emerging economies or in SMEs (Lee 2013). For instance, one common and hidden presumption here is that the firms and other economic actors are already capable of production and innovation, and that the government must simply try to modify the extent of their activities or promote interaction among them. However, the stark reality in emerging countries or in SMEs is that the actors, especially the firms, have extremely weak levels of capability. In the market failure view, the firms are assumed to be capable of conducting R&D, and their only problem lies in their inability to produce the optimal amount. The reasons for a such situation are sought outside the firm, such as in the capital market or risk market. However, the reality in a number of emerging countries is that private firms are unable to pursue and conduct in-house R&D, which they consider an uncertain endeavor with uncertain returns. Thus, a critical matter is how to bring up capabilities of firms in emerging countries or some weak sectors/regions in advanced economies.

Thus, in contrast to the typical argument for government activism based on market failure or system failure, it is also important to consider “capability failure” as a justification for government activism in some specific context (Lee 2013; 2019). Then, effective forms of government activism had better include not the simple provision of R&D funds but various ways to cultivate R&D capability itself. Such direct intervention is important because learning failure happens not only because knowledge is a public good but also the fact that there has been no opportunity for effective learning due to historically inherited conditions or policy failure.

These three types of failure are also helpful to re-interpret the conflicting results on the effectiveness of industrial policy. On the one hand, according to Beason and Weinstein (1996), tariff protection, preferential tax rates, and subsidies did not affect the rate of capital accumulation or total factor productivity (TFP) in Japan from 1955 to 1980. Moreover, nominal tariffs are shown to have had a negative effect on the growth rate of labor productivity and TFP at the sectoral level in Korean industries from 1963 to 1983 (Lee 1996). On the other hand, several studies verify the positive contribution of industrial policy. Shin and Lee (2012), using the same period and sectoral data as Lee (1996), find that tariff protection, especially when combined with export market discipline, leads to the growth of export share and revealed comparative advantage. They also argue that the goal of industrial policy was not productivity at the early stage—as in the 1970s—but output or market share growth. Aghion et al. (2011) also find that subsidies widely distributed among Chinese firms have had a positive impact on TFP and the innovation of new products in the sectors with a high level of competition.

Both of these studies identify competition or discipline as a common precondition for effective industrial policy (Lee 2013). This finding is in line with the findings from detailed analysis of local content requirement policy in auto sectors of Thailand, Malaysia, China, and South Korea (Lee et al. 2021), which argues for the combination of local ownership and both carrots and sticks. Although Malaysia failed in its national brand (Proton) project due to the lack of export-orientation and market discipline, Thailand is a mixed success due to its limited generation of domestic value-added because its auto sectors are owned and dominated by foreign ownership which wanted to conduct core R&D in its home bases. By comparison, China and South Korea have been considered a success due to the eventual rise of locally owned firms that were provided with both carrots of various support and sticks from market competition. In this paper, we will see whether the success conditions of local ownership provided with both carrots and sticks are also applicable to industrial policy for sustainable development.

One way to interpret the diverse outcomes of industrial policy is that the average positive impact of industrial policy might be difficult to verify because the effects tend to appear only in certain conditions, depending upon specific contexts (countries or sectors). Moreover, these studies indicate the significance of the criteria used in assessing the effectiveness of industrial policy.^2^ Given that structural change in an economy is a long-term process, the idea of adopting different policy tools over time or in different contexts is consistent with the reasoning that industrial policy should deal with the various dimensions of capabilities of firms and industries. In other words, different tools are necessary depending on whether the target involves simple operational or production capabilities, investment capabilities, or technological capabilities for sustainable development.

Along this line of thought, an interesting work by Thurbon et al. (2023) proposed the concept of “developmental environmentalism” based on detailed analysis of smart grid and renewable energy sectors in South Korea and China. The book focuses on the delicate mix and sequence of policy tools for creation versus destruction and shows that it is possible for us to both “green” and “grow” our economy, beyond the simple dichotomy of growth versus de-growth. Mathews et al. (2023) in this special issue elaborate further on this concept in the case of offshore wind power sectors in Northeast Asia.

### From tools to new goals: sustainable development

Today, with the need to respond to major social, economic, and environmental challenges, we reach a turning point where new elements come into play pointing to political and social aspects in the elaboration of industrial policy. A gradual widespread rethinking of industrial policy provides arguments on linking industrial development to sustainability goals. Sustainable development should encompass economic and social relationships. However, in the mainstream discourse, invocation of the need to accomplish sustainable development goals neglects the important discussion of how this could be attained in a world of unequal development and uneven production structures. Now governments should act by integrating the public and private spheres as a system that evolves, and industrial policy should find the way to intervene by considering not only economic but also societal goals (Dannreuther and Kessler 2008; Elsner 2014; Ferrannini et al. 2021).

In its recent report on the future of industrialization, UNIDO (2022) refers to megatrends shaping the future of industrial development after COVID-19, rooted in deeper structural shifts related to the process of technological change, socio-demographic transitions, and humanity’s carbon footprint. This context requires a new vision of industrial policy that respects three principles: inclusion, sustainability, and resilience. In that sense, industrial development is defined as “long-term industrialization that drives development along three aspects: creating shared prosperity by offering equal opportunities and equitable distribution of benefits to all; advancing economic competitiveness; and safeguarding the environment by decoupling the prosperity generated by industrial activities from excessive natural resource use and negative environmental impacts.” (UNIDO 2022). This definition implies that industrial development is not merely a question of structural change toward activities of higher value added (Pitelis 2006); it cannot be assessed merely in terms of productivity and economic growth (Criscuolo et al. 2022; Peneder 2017). To address socio-economic and environmental challenges, industrial development should be aligned with societal objectives and long-term qualitative transformations toward an increase in living standards (Criscuolo et al. 2022; Ferrannini et al. 2021; Peneder 2017). In that sense, if the *raison d'être* of industrial policy until now was structural transformation to raise competitiveness and productivity, today this *raison d'être* should be under the condition of sustainability. Actually, the emerging literature on sustainability transitions proposes transformative innovation policy, which may be considered as the third frame, after the two earlier frames of R&D policy and innovation systems, as it focuses on addressing contemporary social and environmental challenges requiring transformative change (Schot and Steinmueller 2018).

## Industrial policy for sustainable development: what should be distinctive about it?

This section intends to identify specific elements that need to be addressed for sustainable industrial development and should be part of the current debate on industrial policy. It argues that a “return-to-normal” (business as usual) approach to industrial restructuring is unsustainable in social, economic, and environmental terms.

Sustainability challenges need public action and government intervention, and industrial policy is a central pillar in the process of structural transformation toward sustainable development. In the race to curb climate change, boost environmental protection, fight global public health emergencies, and confront social inequalities and injustice, industrial policy should announce long-term priorities and performance targets explicitly in line with SDGs.

The renewal of interest in industrial policy, which started in the mid of the first decade of the 2000s and especially in the aftermaths of the 2008 financial crisis, brought in the debate elements with a developmentalist and structural concern, moving away from the focus on resource allocation and the ideological division between state-driven intervention and purely market-based solutions, emphasizing a systemic process considering new challenges as opportunities to rise competitiveness and incomes (Aiginger 2013; Aiginger and Rodrik 2020; Cherif and Hasanov 2019; Rodrik 2014). There were also important contributions including environmental issues into the industrial policy discussion and the need for the greening of industrial policy (Aiginger 2013; Schmitz et al. 2015). Notwithstanding, the discussion in theoretical and methodological terms has been criticized because it retains to a large extent the neoclassical rationale of market failure, government failure, or comparative advantage arguments (Andreoni and Chang 2019; Chang and Andreoni 2020; Peneder 2017; Wade 2018).

As presented in Section 2, a main debate that is relevant when addressing industrial policy tackling sustainability challenges is related to a mainstream approach of market and government failures rationale versus an evolutionary and structuralist approach of system and capability failures and institutional diversity.

What are the new elements that these aspects bring into our reflection on industrial policy? What should be considered differently for sustainability transition? Although at first industrial policy links directly to some of the SDGs (mainly SDG 7, 8, 9, 11, 12, and 17), it can influence all goals. In that sense, any initiatives and actions should be designed with a concern for their implications for all 17 goals. The next subsections discuss a number of issues that should be considered in the formulation of industrial policy in line with sustainable development.

### A focus on production

Sustainable industrial development relates to a process of production transformation. The production and technological paradigm of today has proved to be environmentally unsustainable. Transformation of production patterns towards sustainability must deal with irreversibilities and lock-ins at the organizational and individual levels (technologies, accumulated knowledge, and skills). Commitments and required changes to meet sustainable industrial development goals are faced with high uncertainty and require building production, technological and organizational capabilities.

As already underlined in Section 2, the disconnection between production and innovation that served as an alibi for state intervention during the times of skepticism for industrial policy was based on the strong belief that once a horizontal type of R&D and innovation interventions were put in place, this would translate to higher competitive and economic performance, irrespective of the development stage of the industrial sector or country (Lee 2019). In the mainstream theoretical arguments for industrial policy, there is a neglect of production and the role of learning as a collective and cumulative process embedded in production structures. Learning in production has been acknowledged by many scholars as being of critical importance for structural change and transformation potential of the industrial system (Andreoni and Chang 2019; Best 1990; Chang and Andreoni 2020; Lall 2004; Lazonick 1990; Salazar-Xirinachs et al. 2014) and should be brought at the forefront of the discussion for sustainable industrial development.

The experience of East Asian versus Latin American economies after the 1960s has shown that a capability failure can be a critical determinant because it can undermine the development and diffusion of new technologies, products, and processes and affect the participation of heterogeneous economies in the process of sustainable industrial development (Chang and Andreoni 2020; Lee 2019).

Windows of opportunities in the context of transformation towards sustainability exist at the level of various sectoral systems for those who have built capabilities over the years, still not suffering consolidated irreversibilities, and having set conditions for leapfrogging. Such conditions relate to technological, institutional, and demand factors (Lee and Malerba 2017). Furthermore, structural interdependencies in the productive sector raise vulnerabilities in the context of global crises and require context-specific and coordinated industrial strategies.

Transformation of the production basis calls for investment. Critically important to foster investment for sustainable industrial development is public funding and its directionality, as well as new funding instruments, institutions, and governance modes. The extent of effort to bridge the gap from an old production model to what today seems inevitable but is still difficult to imagine, differs among countries and actors according to their productive and technological base. A new wave of disruptive innovations introducing environmentally sustainable solutions is a prerequisite for a better future but not sufficient because it depends on previous capability building.

Today, many scholars argue for the relevance of a mission-oriented research and innovation policy focused on developing innovative solutions to respond to societal challenges (e.g., health, climate change) (Criscuolo et al. 2022; Larrue 2021; Mazzucato 2018; Robinson and Mazzucato 2019; Wittmann et al. 2020). The effectiveness of these interventions depends on whether they would encompass a wider range of actors and stakeholders of the production and innovation system, change the technology paradigm without reproducing inequalities and economic divergence, and entail ubiquitous use.

### Integration of the demand side

Structural and inter-sectoral interdependencies relate also to demand dynamics along national or global value chains. Scaling-up, improvement of efficiency through integration of the user in the product development process, development of intermediate or complementary goods, and reduction of uncertainty in the development of new technologies are issues influenced also by demand (Criscuolo et al. 2022; Mazzucato et al. 2020).

Greener, healthier, more inclusive growth depends on matching what civil society needs and is able and ready to adopt. Developing solutions for improving nutrition in the world’s poorest regions or ensuring healthy and safer lives for all at all ages requires a coordinated joint strategy focusing on the supply and demand side.

Tackling challenges relating to health, poverty, inequalities, and environmental degradation are social and environmental needs. Interaction of different means with societal need and orchestrating policy instruments to meet sustainability goals encompasses the demand aspect. Furthermore, such integration of the supply and demand dimension should be aligned with the development of new institutions and organizations that will manage the diffusion of such solutions worldwide and with the provision of necessary financial resources.

Institutions and regulations affecting consumers’ preferences or users’ specifications and organizational strategies can induce changes in the production system by triggering innovations or changing the quality of intermediate industrial goods (Maitre-Ekern and Dalhammar 2019; Dolfsma and Mamica 2020). For example, the development of the electric vehicle induced further changes in storage components. Changing individual waste management practices (the demand side) in the context of greening of municipalities by developing the necessary infrastructure, introducing the relevant organizational structures, and altering individual behaviors is a key component in the transformation of the waste management industry (supply side).

Similarly, expansionary macroeconomic policies can be used to create demand in line with sustainable industrial development goals through providing fiscal space for investment and a rise of incomes. Nevertheless, this might not overlook the necessary coordination for developing the new productive, technological, and organizational capabilities aligned to benign growth. High demand as a growth factor has long resulted in a deviation from long-term objectives of sustainable development, as shown by the experience of many emerging countries (China as a world polluter is a case in point). Hence, macroeconomic policy should work in accordance with industrial policy for a sustainable growth path in economic, social, and environmental terms.

### Institutional diversity

Industrial policy is first about structural change. Structural change presents two important features: it is determined by given socio-economic structures and institutions and it is open-ended as once existing structures specify ranges of possibilities, then a path of structural change is open, depending on the actions carried out within those structures in specific historical contexts (Cardinale and Scazzieri 2019).

The process of structural transformation is embedded in an institutional context and should be accompanied by a process of further building and managing institutions. Differences in the development paths but also in the way and rationale of policy interventions at the sectoral, national, or peripheral levels result from alternative combinations of institutions, technologies, and implemented strategies. The co-evolution of technologies and institutions may increase path dependency, and to address sustainability challenges, such path dependencies and resulting lock-ins have to be overcome (Andreoni and Chang 2019; Chang 2011; Srinivas 2020; Thurbon 2014).

Notwithstanding, institutions and institutional change can reduce uncertainty and opportunistic behavior stemming from sustainability challenges, enhance the level of trust for people to engage in societal goals, and ensure sustainable structural transformation. They can also support learning and capability building and affect the adoption and/or diffusion of innovations (for example, changes to the regulatory system, the standards, or the incentives system). Green energy sectors are a case in point as an institutional disruption trying to mitigate climate risk that induced technical progress in many countries (Lema et al. 2020).

The pandemic and the recent geopolitical crisis due to the Ukrainian war have once again highlighted that although shifting away from an unsustainable development process is a universal issue and the challenges are global, interdependencies of choices and the impact of selected options result in divergent and disproportionate changes. Competition regulations, standards, intellectual property regulatory frameworks, intermediate agencies, and financial institutions (such as development banks) cannot align to an a priori best framework, namely, a normative state, but are context specific. This means that industrial policy has to pay attention to the specific features of the sector, country, or region and the interplay of productive and technological change with institutional change and change in societal values. Srinivas (2023) in this special issue elaborates further on this concept in the case of two Indian industry case studies.

### Political economy dynamics

Sustainability challenges, as previously stated, disrupt production and consumption patterns, require new governance and institutional structures (regulations, cultural norms, laws, and organizations). Furthermore, sustainability-focused transition entails a disruption with entrenched incumbent systems exercising policy influence but also interaction with emerging coalitions eventually supporting the transition process (Roberts et al. 2018). SDGs address challenges such as poverty, inequalities, inclusion, health safety, and environmental degradation, which entail political economy dynamics.

Power relations are disrupted or further consolidated as new windows of opportunities open for new entrants or incumbents that diversify in new activities, redeploying their resources and capabilities. For example, firms producing or using polluting energy turn into green energy, “brown firms” exit and “green entrants” are favored, countries (India investing in green hydrogen is a typical example) seek strategic autonomy through environmental conversion, and a new set of winners and losers emerges (Mathews et al. 2023; Sandbu 2021). Such transformations and shifts in the geopolitical model create new types of politics. Conflicts among social groups, firms, countries, and forms of capital are intertwined with changes in the institutional context and require a new societal vision reconciling sustainability and development (Chang and Andreoni 2020; Ferrannini et al. 2021; Peneder 2017; Roberts et al. 2018). The emergence of new winners and losers because of transformation, and the existence of different sources of conflicts may entail political capture hindering industrial policy to serve sustainability goals. This is particularly challenging for countries with low institutional quality, where lobbying groups and competing interests alter industrial policy goals.

These issues highlight the need for an industrial policy that considers the question of democratization and just transition. Therefore, attention should be paid to elements that improve economic performance (productivity and competitiveness) while reducing inequalities and directing transformation to collective interests conceived in terms of societal objectives of sustainability and inclusion (Cardinale and Scazzieri 2019; Ferrannini et al. 2021). Furthermore, civic engagement, participatory formulation of industrial policy, and consensus at different levels are important prerequisites to fulfill the SDGs (Aiginger 2013, 2015; Ferrannini et al. 2021; Mamica and Dolfsma 2022; Rodrik and Sabel 2022; Tagliapietra and Veugeler 2020).

Hence, whether transformation will favor incumbents and reproduce relations of power or will occur complying with inclusion and widespread participation are issues to be considered. If the latter is undermined, then, it is mostly probable that a situation with uneven environmental and social repercussions of economic activity will pertain.

### Coordination—management of conflicts and complementarities

In different contexts (regional, national, or global), system dynamics (resulting from interdependencies of structural, institutional, and political elements) might not coincide with what is collectively conceived as sustainable development (Cardinale and Scazzieri 2019). Depending upon the content of sustainable development and on the specific mission to be implemented, diverse underlying specific goals relate and influence the direction of industrial development. Hence, multiple policy areas are implicated with stronger or weaker complementarities, and coordination across policies, policy instruments, or various stakeholders is required (Criscuolo et al. 2022; Larrue 2021). Complementarities shape sector dynamics; for example, the diffusion of renewable energy sources is related to advancements in battery technologies or digital transformation of energy providers. Interdependence with macroeconomic dynamics should be considered because, especially in less developed economies, they might constrain the fiscal space for implementing structural change addressing sustainability challenges. There might be a vicious cycle of weak productive and technological capabilities that undermine the industrial system’s resilience that in turn may reduce strategic autonomy in the management of the main macroeconomic instruments to achieve economic performance in terms of competitiveness, income, or creation of quality jobs. Coherence and coordination can reduce uncertainty resulting from high interdependencies.

These issues point to the importance of public management in understanding the system’s dynamics, creating conditions for collective action, and coordinating highly heterogeneous actors and industrial activities. They also imply that new capabilities and skills are required for the attainment of public goals.

Implementation of industrial policy for sustainable development is a process encompassing various stakeholders from the private and public sectors and civil society. As Rodrik argued (2014), there is a need for continuous collaboration and dialog that would capitalize on feedback from sequential policy implementation.

Last but not least, as previously discussed, there is high heterogeneity across countries in terms of capabilities, opportunities, and needs. Especially for developing and middle-income countries, the lack of productive, technological, and organizational capabilities; public management deficiencies; and difficulties in mobilizing financial resources renders implementation of sustainable industrial development extremely complex and controversial and, thus, coordination issues more pressing. Policy measures targeting one SDG could undermine the achievement of others. For example, high taxes in the context of the emission trading system on energy consumption to meet the goal of curbing climate change might constrain the goals for sustainable industrialization, innovation, reducing poverty and inequalities, and promoting social inclusion, and, especially at a global scale, the environmental sustainability of less developed countries because of carbon leakage.

### Evaluation of industrial policy for sustainable development

Although there is no consensus on an industrial policy framework for sustainable development so far, there is an increasing interest in sustainability goals, and more and more scholars advance arguments that industrial policy should go beyond productivity growth and competitiveness, emphasizing economic, social, and environmental goals (Aiginger 2013, 2015; Aiginger and Rodrik 2020; Criscuolo et al. 2022; Ferrannini et al. 2021; Peneder 2017; UNIDO 2022). In parallel, the discussion of the consequences of climate change such as drought and extreme weather events and their costs is becoming increasingly important in the public debate. A question arising then is as follows: based on what criteria should industrial policy be assessed? Performance of industrial systems at the regional, national, or international levels should be monitored and evaluated against SDG indicators to assess whether economic, social, and environmental goals are achieved.

With that in mind, implementation of industrial policy requires evidence-based selection of strategic targets, a monitoring and evaluation framework and new mechanisms ensuring accountability and transparency. These are critical conditions to deal with political capture risk, to ensure that state investment serves particular targets for sustainable development, creating long-term value and aligning private and public interests.

Following this reasoning, technologies should not be evaluated merely on the basis of productivity, competitiveness, or innovation potential in the long run, but on the basis of whether they serve long-term societal goals. Examples include reducing greenhouse gas emissions or reducing water consumption in technological processes. This would mean that many countries competing based on price competitiveness would face important deadlocks. However, this issue relates to the discussion on capability failures presented in Section 2.2.

Furthermore, in a world of complex and increasingly interdependent systems, the ability to absorb shocks and avoid failures depends more on redundancy than efficiency as the latter increases in many cases vulnerability. Lack of redundancy and risk management based on selecting the leanest possible operations might prove to be ruinous in some cases for economic and social systems. In the same vein, building system resilience emphasizes the importance of recovery and adaptation in the aftermath of a crisis, keeping in mind that future threats and their effects cannot be adequately predicted and measured. If we address sustainable challenges with this mindset, we should consider, for example, the climate disruption to aggravate and be combined with new pandemics; thus, industrial systems should be able to anticipate, absorb, recover from, and develop new solutions to emergencies (Criscuolo et al. 2022). Monitoring, learning from shocks, reviewing, and adapting (what a typical control function would encompass) are a critical element for shaping industrial policy for sustainable development.

### Discussion

This section emphasized that to address sustainable development, there is need for a novel way of reasoning regarding industrial policy. A revamping of industrial policy first relates to a political process that ideally should result in a consensus on a mission for sustainable development (to save the planet, reduce poverty and inequalities, and advance wellbeing). Such a consensus needs the identification and involvement of all stakeholders and implies a system of policies that would target SDGs. At the roots of such a process, there are contradictions and controversies due to the exercise of power and a central stake: the distribution of gains and losses. The way the political process interplays with the institutional setup and productive structures implicitly or explicitly influences the direction and outcome of transition to a more sustainable mode of economic activity.

A variety of policies intervene at different ontological levels: individual, organizational, or system. Policies targeting firms and other types of organizations, or specific industries, technologies, or clusters, or those setting the framework with rules and institutions and interventions are all part of a system of policies to foster structural change. Deriving from a political consensus, industrial policy is a main component of this system of policies and should align with the overall vision of sustainable development. The critical issue is to put in place strategies that will link its *raison d'être* to building capabilities and developing institutions especially where voids in learning processes and institutional settings work against equality, democracy and sustainability.

In this context, orchestration of policy initiatives should encompass building productive, technological, and organizational capabilities; fostering demand and institutional transformation; and mobilizing and directing financial resources to meet SDGs. Once there is an agreement on specific goals—examples include that polluting technologies harming the environment should be substituted with cleaner solutions or that healthy and safer lives should be ensured for all at all ages—then, all specific policies at different levels of intervention should align with this specific target. In that case, there is no space to talk about interventions justified with reference to mainstream “failures,” but policies defining the context within which economic activities will take place or emerge in compliance with societal needs (Peneder 2017).

**Fig. 1:**
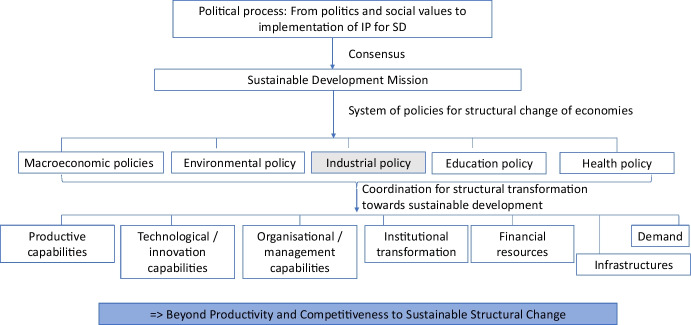
Industrial policy as a process to sustainable industrial transformation

Figure 1 schematically represents the process of how we could get from the level of agreeing on the general social values to the implementation of specific industrial strategies translated into specific objectives to meet sustainable industrial development. The ultimate goal is urgently required to go beyond productivity and competitiveness toward consideration of ecological, social, and economic goals taken together for a long-term rise in life standards. Genuinely, the process is not linear but co-evolutionary, involving interconnections of various elements at different levels. Furthermore, it remains open and dependent on the political and socio-economic context in which industrial policy is implemented, whether a consensus could be attained, and whether the underlying sustainable development objectives of inclusion, prosperity, equitable distribution of benefits, and environmental protection could be reached.^3^

## A brief summary of special issue papers and the major issues

This special issue draws together a number of papers addressing some of the issues developed in the previous section. These papers have been selected after an open call launched in April 2021, followed by a rigorous review process.

The energy transition from fossil fuels to renewable energy sources is becoming a reality through private sector investment as well as targeted public support policies. An example of this type of transition is the dynamic development of offshore wind power (OWP) in Northeast Asian countries such as China, Japan, South Korea, and Taiwan (Mathews et al. 2023). The involvement of the governments of these countries, as well as their specialized agencies, in the promotion of OWP as part of a broadly understood new generation of industrial policy can be discussed in the context of developmental environmentalism. The efforts of the public sector are supported by private actors, including fossil fuel companies, which see OWP as an opportunity to contribute to the green transition, such as by finding new uses for floating oil platforms. All actors in the energy industry are becoming increasingly aware of the necessity to engage in this unique sort of Schumpeterian creative destruction. Companies with significant financial and human resources that have traditionally been involved in the exploitation of fossil fuels are increasingly keen to invest in renewable energy sources. They are also learning from one another about how to build capacity with public monies and construct hybridized industrial ecosystems (Kim 2019). However, the rate of private investment is considerably affected by state investment support policies for OWP. The policy goal is to take the lead in the competition between Northeast Asia, the European Union, and the USA.

To mitigate the negative effects of climate change, it is critical to prevent the degradation of forest resources, which serve as a natural carbon store, apart from taking critical steps to shift away from the use of fossil fuels and toward the use of renewable energy. The study of the Amazon Fund, dedicated to the conservation of the Amazon forest, comprising the period from 2008 to 2021, is an example of a systemic solution in this area (Ferraz et al. 2023). Its success was made possible by a number of novel methods, including multi-stakeholder governance, donor-based pay-for-performance funding, and non-reimbursable project financing from the Brazilian Development Bank. First, a multistakeholder committee, the Amazon Fund Guidance Committee, was set up to oversee strategizing, oversighting, and monitoring the investments made. Second, donations were based on a pay-for-performance basis: if deforestation was avoided, donations could be raised and vice versa. Monitoring was to be conducted by an independent technical committee, using an existing reliable satellite monitoring system. Third, financial resources were to be centrally managed by the Brazilian Development Bank and operationalized through non-reimbursable finance where no obligation existed to return the loaned resources or to provide collaterals. Such an optimization of existing resources is consistent with the goals of the green industrial policy (Mathews 2020). With Bolsonaro gone, there is hope that the new government by Lula da Silva will restore the Fund’s role in protecting the Amazon Forest.

Klebaner and Voy-Gillis (2022) analyze the challenges faced by industry in the context of energy transition on the one hand and reducing its negative environmental impacts on the other. In the authors’ view, France’s current industrial policy instead of promoting change leads to stagnation because of conflicts between aims, tools, and stakeholders, as well as the high level of complexity. In practice, short-term business strategies predominate over genuine efforts to chart a radically new course. The emphasis of a new industrial policy should be on qualitative rather than quantitative growth not least due to the diminishing contribution of industry to France’s GDP, which was over 30% in the late 1970s but dropped to only 10% in 2019. The COVID-19 pandemic, which demonstrated in stark terms the extent to which the country’s economy was dependent on imports from China, particularly key products in the context of combating the pandemic such as masks, drugs, and medical equipment, marked another key point in the discussion of industrial policy change in France after the 2008 crisis. As a result, the issue of reindustrialization has been reintroduced into public discourse, with mechanisms such as state-guaranteed loans (PGE) and short-term working arrangements for employees being considered to stimulate industrial development. However, France’s industrial strategy is unstable, which continues to cause uncertainty, as conflicts between aims, tools, and stakeholders lead to a situation that short-term business strategies predominate over genuine efforts to chart a radically new course of industrial policy.

Despite the globalization of the international economy, local guidelines, norms, and laws still have a considerable impact on business activity. Srinivas’ two health sector cases from India exemplify the problem of institutional variety (Srinivas 2023). The first case, Ayurveda, is a traditional medicine with manufacturing elements of different scales, messily situated amidst “traditional,” “informal,” “cottage,” or “home-based” labels produced by micro-, small-, and medium-sized firms operating without standardization protocols or certification. From the perspective of Western medicine, the high level of interest among the Indian public in the treatment options offered by Ayurveda contributes to tensions with other pharmaceutical and biotechnology companies. These are due to the regulatory responsibilities being dispersed among the various actors at the domestic level and to the lack of supervision over the collection and trade of raw materials sourced by the company. The lack of market regulations is not incompatible with the rapid growth of Ayurveda, which is fueled by lifestyle health choices and growing distrust of mainstream medicine in Indian society. The situation of this health industrial system may also be examined in the context of the debate on the role of regulation in ecological sustainability. In the second case, the medical oxygen industry came under close scrutiny in India, as it did in many other countries, during the peak of the COVID-19 pandemic. The availability of specialized liquid medical oxygen storage tankers on Indian Railways and the coordination of centralized government regulatory offices with decentralized parties kept the supply market from collapsing. The regulated market for the manufacture and distribution of oxygen to a primarily industrial clientele allowed the output required in hospitals to rapidly rise through defense–civilian partnerships.

The two Indian cases support the argument that meso- and micro-heterogeneity better combine ecological, health, and industry concerns and that rules and standards set by industrialized economies are sometimes not well-suited for ecology and climate risks. State coordination of actors and a combination of distinct levels of institutional variety are needed for sustainable industrial policies.

Critics of industrial policy argue that it tends to be overly focused on economic growth objectives to the detriment of environmentally sensitive and socially inclusive spatial economic development. Obeng-Odoom (2022) addresses this issue in the context of rising urban inequalities and ecological problems affecting cities and regions. According to him, a well-designed industrial policy should enable the recovery of unearned revenues that have been socially created but privately appropriated by an absentee class abroad. These monies should have then been used to build state capacity to address ecological concerns and reduce urban inequities. Obeng-Odoom illustrates the problem of industrial policy, using the case of Ghana, a country that is fairly well developed by African standards that has a strong oil and natural resource industry, with the twin cities of Sekondi and Takoradi serving as industrial hubs. These cities are a good illustration of both the potentials and limitations of Ghana’s industrial policy: rapid economic growth has not resulted in the environmentally conscientious and inclusive economic development that was expected, and worker satisfaction levels have remained low, whereas the profits were monopolized by TNCs and their owners abroad.

Green industrial policy also includes assisting businesses that boost productivity by utilizing waste products, thus eliminating the need for their disposal. Enterprises that generate such waste can subsequently minimize their environmental impacts, reducing and internalizing their environmental costs, which is a key component of the green growth idea (Rodrik 2014). This is known as industrial symbiosis, in which one company’s waste or by-product is used as a raw material by another. Industrial symbiosis is especially appealing to developing countries because it does not necessitate large capital inputs. In their analysis of three Ugandan cases of industrial symbiosis in action, Buda and Ricz (2023) discuss the potential for green development and state intervention meant to foster it. The first case is a start-up established by students at Makerere University in 2018, which manufactures biodegradable plates using an invasive plant species that blooms in Lake Victoria. The second case provides compost, an animal husbandry by-product, to neighborhood clients, and the third case is a non-profit firm that produces handwoven textiles using waste cotton yarn, off-cut fabrics, and vegetable fibers from banana stems. In all three cases studied, resource efficiency is as important as productivity.

Interviews with managers of these firms revealed their hopes for the development of technical infrastructure, tax cuts, microloans, and contributions to research and development. These aspects of support should thus be considered one requirement of success when setting the goals of green industrial policy. Overall, these experimental cases of industrial symbiosis in Uganda show that realizing environmentally friendly business in the context of a low-income economy in Africa is not impossible, but the binding issue is whether tangible and intangible resources are sufficient to support these experiments so that they may survive in the competition in the market against conventionally made goods.

## Summary and concluding remarks

Until the 2008 financial crisis, industrial policy remained outside mainstream economics literature and may have even been outside the realm of innovation support, despite its undeniable triumphs in East Asia in the last decades of the twentieth century. Support for industries at the national level has become accepted as a result of the post-pandemic breakdown of supply chains, including an especially severe one in the microprocessor industry and mounting economic tensions between China and the USA. This was followed by a growing recognition among governments of the significance of public R&D spending. The numerous strategies to cultivate R&D capability appear to be as significant as the magnitude of this expenditure stream. As was previously demonstrated, the effectiveness of the industrial policy tools not only varies over time but also depends on specific contexts (countries and sectors).

The increasingly acute consequences of the climate crisis, the recovery from the COVID-19 pandemic, and the energy crisis caused by Russia’s invasion of Ukraine have put industrial policy back in the spotlight of political and public debate. Successfully addressing socio-economic and environmental challenges requires a new vision of industrial policy that upholds three principles: inclusion, sustainability, and resilience. A shift in the industrial production paradigm must address issues other than technology, such as poverty, inequalities, inclusion, health safety, and environmental degradation.

The success of industrial policy for sustainable development requires a shift from viewing profits as individual gains realized by economic actors to a perspective of social and environmental gains. These can be defined by the neutrality of the environmental impact of economic activities (including, in particular, greenhouse gas emissions) and the positive effects on the local community.

The papers in this issue examine how different countries implement industrial policy for sustainable development from a variety of perspectives. They highlight convergent and divergent forces and processes, which interplay in sustainability transitions. They give insights from different aspects to understand the challenges entailed in the implementation of industrial policy for sustainable development, combining ideas from evolutionary and institutional economics.

As the not-so-optimistic example of France shows (Klebaner and Voy-Gillis 2022), the current industrial policy, rather than supporting energy transition, leads to a standstill due to its complexity and conflicting goals, tools, and stakeholders. Furthermore, as Ghana’s Sekondi–Takoradi case shows, the growth-centered industrial policy without local ownership has not generated the expected results in terms of urban inclusion and environmental protection (Obeng-Odoom 2022).

The extent and pace of implementation of industrial policies for sustainable growth at the level of individual countries are still determined by different levels of institutional variety. The two health industry cases from India functioning under COVID-19 pandemic conditions serve as a good illustration of this issue (Srinivas 2023). Utilizing waste materials in the production process in accordance with the principles of green industrial policy, a practice pursued by two Ugandan enterprises, is one of the still-unappreciated opportunities to reduce the detrimental impacts of industry on the environment (Buda and Ricz 2023). Efforts to increase the amount of greenhouse gases naturally absorbed by healthy ecosystems should not be disregarded either. The Amazon Fund, which successfully prevents deforestation in the Amazon Forest through a range of creative solutions, is discussed as an example of good practice in this respect (Ferraz et al. 2023).

The paper of Mathews et al. (2023) by looking at the emergent offshore wind power industry argues that transformation for sustainable development entails competitive dynamics involving emerging and incumbent players, the latter leveraging their accumulated experience and capabilities. In Northeast Asian countries, state-led strategies for industrial transformation, although different to some extent, have in general terms ascribed to a developmental environmentalist strategy, with state agencies playing a central role in the green transition.

In the market failure logic, the need for state activism arises due to the gap in social and private returns associated with externalities from certain strategic sectors. Now, in the context of sustainable development, the need for state activism arises due to the similar gap and externalities associated with environment sectors, technologies, and institutions. But, one learning from special issue papers is that the nature and tools of intervention or industrial policy might be different. Specifically, we may note a subtle difference in the success condition for new industrial policy for sustainable development and that for traditional industrial policy. Drawing upon Lee et al. (2021), Section 2.2 points out the local ownership provided with both carrots and sticks as the success conditions for traditional industrial policy. Now, transition to sustainable development also seems to require a right mix of carrots and sticks to boost new sectors but an additional requirement is to strike a right balance of creation versus destruction in Schumpeterian creative destruction, for instance, the creation of new renewable businesses and the destruction of old fossil-fuel businesses. The old fossil-fuel based sectors are not just destroyed but their experiences and competences can be utilized, and they themselves may switch to producing renewables.

Furthermore, because institutional diversity and the risk of capture can result in very distinct economic, social, and environmental effects, consideration of heterogeneity at country and sector levels, and coordination of vested interests by forming a right governance structure are essential ingredients for sustainable industrial policies, as shown by the case of industrial policy in France and the two industry cases in India. By contrast, the case of the Amazon Fund is indicative of three success elements: multi-stakeholder governance, donor-based pay-for-performance funding, and non-reimbursable project financing. These three conditions are comparable to the success conditions for traditional industrial policy. First, stakeholder governance amounts to local ownership, and this element can then be summarized as “local ownership and accountable governance.” Second, project financing plays the role of carrots, whereas pay-for-performance has the role of minimum sticks. Then, the success conditions comprising both traditional and new industrial policy can still be summarized as local ownership and accountable governance, provided with both carrots and sticks. This may serve as a good formula for any project for sustainable development, especially those involving international dimensions and stakeholders. The case of OWP in northeast Asia also seems to meet this success condition.

This principle can also be applicable in assessing other cases treated in the special issue. For instance, the problematic case of urban development driven by the oil industry in Ghana can be criticized in terms of the lack of local ownership of oil resources and production, which has led to all profits being monopolized by the absentee class or foreign-based land/resource lords (Obeng-Odoom 2022). In comparison, the mixed success and the constrained growth of three cases of industrial symbiosis in Uganda (Buda and Ricz 2023) can be attributed to the lack of effective carrots (pro-active support by the government) whereas they are exposed to discipline from market competition (sticks).

This discussion implies an interesting hypothesis that although traditional industrial policy and new industrial policy are different in terms of their goals and tools, both may be assessed in terms of the same success conditions of responsible ownership and governance provided with both carrots and sticks. The cases collected in this special issue also seem to imply that any success of industrial policy for sustainable development would require handling well all the three types of failure, such as market, system, and capability failures although specific weights among these three might be different in different contexts. In other words, it necessitates building capabilities of involved actors and coordinating collaborative actions of agents over the globe, besides providing socially optimal incentives to reflect positive externalities of global public goods.

Overall, the shift of the focus of industrial policy is consistent with the shift of the role of the state, from developmental states (Johnson 1982; Thurbon 2014), as in the early East Asia, to entrepreneurial states (Mazzucato 2011) in the Western advanced economies, and finally to environmental states promoting sustainable development at a global scale. The switch to environmental state may be not exclusive. Rather, what each country needs may be some combination of all of these three states where the right balance can be different depending upon a country’s level of development and the political economy of involved actors. In developing or emerging economies, a viable combination may be “developmental environmentalist,” as shown by Mathews et al. (2023) and Thurbon et al. (2023). For advanced economies, it may be “entrepreneurial environmentalist.”

In the meantime, recently since the rising tension between the USA and China, another role of the state has been given attention and needs to be studied in the future: the “national security state” which was first proposed by Weiss (2014). Weiss attributes the US capacity for transformative innovation to the strength of its national security state, which is a complex of agencies, programs, and hybrid arrangements that has developed around the institution of permanent defense preparedness and the pursuit of technological supremacy.

## Footnotes

1 Production stage means simply the process of making goods and services with hired workers.

2 For example, while an earlier study finds no effect of industrial policy on productivity (Lee 1996), productivity has become an important criterion only since the late 1980s in South Korea. Only after the Korean government shifted its policy tools from tariffs to research and development (R&D) subsidies as well as joint R&D, such new tools for enhanced innovation capabilities are shown to have led to Korean firms’ productivity catch-up with Japanese firms from 1985 to 2005 Jung and Lee (2010)

3 An interdisciplinary research approach would further advance our understanding on the political and social embeddedness of industrial policy in different historical contexts. We thank an anonymous reviewer for his comments on the question of power and social groups’ diverging interests.
